# Exploring the role of indoor microbiome and environmental characteristics in rhinitis symptoms among university students

**DOI:** 10.3389/frmbi.2024.1277177

**Published:** 2024-02-16

**Authors:** Xi Fu, Aga Shama, Dan Norbäck, Qingsong Chen, Yun Xia, Xin Zhang, Yu Sun

**Affiliations:** ^1^ Guangdong Provincial Engineering Research Center of Public Health Detection and Assessment, School of Public Health, Guangdong Pharmaceutical University, Guangzhou, China; ^2^ Occupational and Environmental Medicine, Department of Medical Science, University Hospital, Uppsala University, Uppsala, Sweden; ^3^ Institute of Environmental Science, Shanxi University, Taiyuan, China; ^4^ Guangdong Provincial Key Laboratory of Protein Function and Regulation in Agricultural Organisms, College of Life Sciences, South China Agricultural University, Guangzhou, Guangdong, China

**Keywords:** university dormitory rooms, 16S rRNA, proteobacteria, rhinitis, settled air

## Abstract

**Introduction:**

Rhinitis is one of the most prevalent chronic respiratory diseases worldwide. There is emerging evidence suggesting that the indoor microbiome may contribute the onset and exacerbation of rhinitis symptoms, but comprehensive studies on this topic remain scarce.

**Methods:**

In this study, we assessed the microbiome assemblage of settled air dust collected in Petri dishes in 86 dormitory rooms of Shanxi University, China using 16s rRNA sequencing. A self-administered questionnaire, including questions about rhinitis symptoms and personal information, was completed by 357 students residing in these dormitories. Logistic and linear regression model was applied to examine the associations between environmental characteristics, indoor microbiome, and rhinitis.

**Results:**

The most abundant genera in the dormitories were *Ralstonia* (15.6%), *Pelomonas* (11.3%), *Anoxybacillus* (9.3%) and *Ochrobactrum* (6.2%). Taxa richness in the class of Actinobacteria and Fusobacteriia was negatively/protectively associated with rhinitis (p<0.05). Six bacterial genera, including those from Actinobacteria (*Actinomyces*), Fusobacteriia (*Fusobacterium*), and Bacteroidetes (*Prevotella* and *Capnocytophaga*), were negatively/protectively associated with rhinitis. Conversely, seven genera, predominantly from Alphaproteobacteria and Betaproteobacteria (*Sphingomonas, Caulobacter*, uncharacterized *Caulobacteraceae* and *Comamonadaceae*), were positively associated with rhinitis. Living in higher floor level and higher indoor PM_2.5_ concentrations were associated with a higher abundance of taxa potentially protective against rhinitis and a lower abundance of taxa potentially increasing the risk of rhinitis (P<0.01). However, having curtain indoor and higher indoor CO_2_ concentrations were associated with a lower abundance of taxa potentially protective against rhinitis and a higher abundance of taxa potentially increasing the risk of rhinitis (P<0.01).

**Discussion:**

This study enhances our understanding of the complex interactions between environmental characteristics, indoor microbiomes, and rhinitis, shedding light on potential strategies to manipulate indoor microbiome for disease prevention and control.

## Introduction

Rhinitis, characterized by nasal symptoms such as congestion, runny nose, and sneezing, is one of the most prevalent chronic respiratory diseases worldwide, affecting up to 30% of the global population (Bousquet et al., 2008; Savouré et al., 2022). The health impact of rhinitis extends beyond the characteristic symptoms, often leading to substantial impairment in the quality of life. It is associated with sleep disorders, cognitive impairment, and a significant reduction in work and school productivity (Walker et al., 2007). Furthermore, rhinitis is often a comorbidity in patients with asthma, which can complicate disease management and exacerbate health outcomes (Brożek et al., 2017). Given its high prevalence and substantial impact on health and wellbeing, understanding the factors contributing to rhinitis is of utmost importance.

Rhinitis research has been extensive, yet the etiological landscape of the disease remains complex due to its multifactorial nature (Hellings et al., 2017). Genetic predisposition plays a role in rhinitis, with several candidate genes and single nucleotide polymorphisms (SNPs), such as HLA-D, TCR, and CD14, reported to be associated with the condition (Wang, 2005). Environmental factors, notably indoor and outdoor air pollutants, are reported to play a significant role in the onset and exacerbation of rhinitis symptoms (Chen et al., 2001; Norbäck et al., 2019; Lu et al., 2020; Lu et al., 2022). Among these, indoor allergens such as dust mites, pet dander, and mold spores are frequently implicated in allergic rhinitis (Zock et al., 2007). Lifestyle factors, including tobacco smoking and diet, can also influence rhinitis risk and severity (Guerra et al., 2002).

The indoor microbiome, the diverse community of microorganisms inhabiting our indoor environments, has emerged as a significant factor in the context of respiratory diseases, including rhinitis. These microbial communities, present in indoor dust and air, can influence our health either directly, via inhalation and exposure, or indirectly through their metabolic activities (Chen et al., 2023; Zhang et al., 2023a). A few recent studies reported several potential protective (*Prevotella*, *Lactobacillus iners*, and *Dolosigranulum*) and risk indoor microorganisms (*Collinsella* and *Geodermatophilus*) (Fu et al., 2021c; Sun et al., 2022a; Fu et al., 2023a). However, there are still several major limitations in this research area. First, compared to the substantial research exploring the association between indoor microbiome and asthma (Ege et al., 2011; Lynch et al., 2014; Kirjavainen et al., 2019; Fu et al., 2021a; Fu et al., 2021b; Fu et al., 2021d; Fu et al., 2022; Sun et al., 2022b), there is a relative dearth of such studies for rhinitis (Sun et al., 2022a; Fu et al., 2023a; Sun et al., 2023). Second, most previous research has characterized indoor microbiomes by assessing vacuum dust collected from floors and tables. This approach may not accurately reflect the microorganisms to which individuals are typically exposed at the breathing level, as some of the floor microorganisms may not be resuspended to the height of human breath (Hyytiäinen et al., 2018). Thirdly, environmental characteristics have been neglected in previous studies. This omission hinders our understanding of potential factors that could affect the abundance of rhinitis-related microbial taxa and consequently impedes the potential application of environmental remediation and intervention strategies in indoor environments. Overall, there is a need to further explore the complex relationships between indoor microbiome and rhinitis, in order to advance our understanding of the disease and to open up new avenues for prevention and treatment.

To address these issue, our study aimed to investigate the relationship between the indoor microbiome and rhinitis by assessing the microbial composition of settled air dust collected from 86 dormitory rooms at Shanxi University, China. To address this limitation of floor vacuum sampling, our methodology involved using open Petri dishes hung at the breathing level for several days in the dormitory rooms, offering a more representative sampling of the airborne microorganisms that individuals are likely to inhale. Utilizing 16s rRNA gene sequencing, we identified the predominant bacterial genera and examined their associations with the occurrence of rhinitis. Our findings reveal a diverse bacterial assemblage, and importantly, unique associations with rhinitis. This study also examined the impact of environmental characteristics on these rhinitis-associated bacteria, further enriching our understanding of the interplay between environmental characteristics and indoor microbiome. We anticipate that our findings will contribute to the growing body of knowledge in this field and could potentially inform future preventive and therapeutic strategies for rhinitis.

## Methods

### Study design

Dust samples were collected from 86 rooms, which were randomly selected from the several hundred available dormitory rooms at Shanxi University, Taiyuan, China. These 86 rooms spanned ten different dormitory buildings, with 8-10 rooms chosen from each building to ensure representation from various buildings and different floor levels. Building 1-5 were from a new campus (LD), and building 6-10 were from an old campus (WY) of Shanxi University. The two campuses were 1.5 kilo meters away. The sampling was conducted in November and December 2013. Settled air dust was collected by two open-up Petri dishes 1.2-1.5 m high above the floor for seven days. All 86 settled air dust samples were qualified during DNA extraction and library preparation process and performed amplicon sequencing. The study was approved by the Medical Ethics Committee of Fudan University, Shanghai, China, and all participants gave their informed consent.

### Collection of dust sample and environmental characteristics

Two open Petri dishes was laid on a flat surface around 1.2-1.5 meters high for seven days to collect the settled air dust. All dormitory rooms were sampled in November and December 2013. After sampling, the Petri dishes were washed by 2ml PBST and stored in Eppendorf tubes. The resulting PBST wash solution, containing the dislodged microorganisms from the settled air dust, was transferred to Eppendorf tubes and stored at -80°C for subsequent DNA extraction.

We collected a total of 16 environmental characteristics through inspection, measurement, and the use of questionnaires. Characteristics displaying co-linearity (r > 0.4) were excluded from our data set, which included the curtain size, roof type, and window to floor area ratio. Similarly, self-reported characteristics such as floor cleaning frequency and method were not included due to the risk of recall bias. Our regression analyses, therefore, incorporated eight environmental characteristics, including building age, floor level, wall surface type, having curtains, curtain cleaning frequency, having plants indoor, and indoor concentrations of CO_2_ and PM_2.5_. All environmental features were meticulously inspected or measured by trained graduate students. Indoor PM_2.5_ and CO_2_ concentrations were determined using the Dusttrak II Aerosol monitor and the Q-TRAK IAQ monitor respectively, both from TSI Incorporated, St. Paul, MN, USA. The PM2.5 monitor was calibrated using data from the closest ambient air monitoring station for accuracy.

### Health data collection

A self-ministered questionnaire was sent to the occupants of the dormitories, and 357 students participated (97.3%). The questionnaire included questions on rhinitis and personal information like gender and smoking habit. The questions about rhinitis were adapted from the European Community Respiratory Health Study (ECRHS). This classification has undergone rigorous validation in various studies and has been broadly employed in epidemiological investigations (Zhang et al., 2013; Sun et al., 2022a; Fu et al., 2023a). Participants were asked two questions to assess their recent health conditions. The first question pertained to the frequency of rhinitis symptoms, such as a stuffy or runny nose, experienced over the past 12 months. Also, these rhinitis symptoms occurred when you do not have cold or upper respiratory infections. The available response options were: (A) “Yes, everyday”, (B) “Yes, 1–4 times per week”, (C) “Yes, 1–3 times per month”, and (D) “No, never”. Those who selected either (A) or (B) were classified as having rhinitis symptoms.

### Bacterial DNA extraction and amplicon sequencing

Total genomic DNA was extracted by E.Z.N.A. Soil DNA Kit D5625-01 (Omega Bio-Tek, Inc., Norcross, GA, USA), followed the manufacturer’s instruction. Bead beating and spin filter techniques were used for DNA extraction. Extracted DNA quantity and quality were evaluated by NanoDrop ND-1000 spectrophotometer, agarose gel electrophoresis and Microplate reader (BioTek, FLx800). Negative control with reagent was added to evaluate laboratory microbial contaminations in the agarose gel electrophoresis. Forward primer 338F (ACTCCTACGGGAGGCAGCA) and reverse primer 806R (GGACTACHVGGGTWTCTAAT) were chosen for the 16s rRNA gene V3V4 region amplification. Sample specified 7-bp barcode sequences were incorporated into the multiplexing step. PCR reagents included 1 μl (10 μM) of forward and reverse primers, 2 μl DNA template, 5 μl Q5 reaction buffer, 2 μl (2.5 mM) dNTPs, 5 μl Q5 High-Fidelity GC buffer, 0.25 μl Q5 High-Fidelity DNA polymerase and 8.75 μl of ddH_2_O. PCR started with a 2 min 98°C denaturation, followed by 25 cycles of 15s 98°C denaturations, a 30s 55°C annealing and a 30s 72°C extension process, and the whole PCR process ended with a 5 min extension at 72°C. PCR amplicons were purified by Agencourt Beads (Bechman Coulter, Indianapolis, USA) and quantified by PicoGreen dsDNA Assay Kit (Invitrogen, CA, USA). In total, 169 samples were successfully amplified and further sequenced by Illumina MiSeq platform with MiSeq Reagent Kit v3. DNA extraction and multiplexed high-throughput sequencing were conducted by Personalbio (www.personalbio.cn). The sequencing data have been made available in Qiita under the study ID 12841 (https://qiita.ucsd.edu/study/description/12841).

### Bioinformatics and microbiome analysis

Raw sequences were extracted according to the barcode information and filtered with the following criteria: minimum reads sequence length > 150 bp, average Phred score > 20, contained no ambiguous bases and no mononucleotide repeats that > 8 bp (Gill et al., 2006). Chimeric reads were removed by USEARCH (v5.2.236) (Edgar, 2010), and paired-end reads were assembled by FLASH v(1.2.7) (Magoc and Salzberg, 2011), with minimum 10 bp overlapping between forward and reverse reads without mismatches. The assembled high-quality reads were clustered into operational taxonomic units (OTUs) at 97% sequence similarity threshold by UCLUST (v5.2.236) (Edgar, 2010). A representative sequence from each OTUs was selected to blast against the Silva ribosomal RNA database (release 115) (Quast et al., 2012) to acquire the taxonomic information by using the best hit. The following analyses were conducted in Quantitative Insights Into Microbial Ecology (QIIME, v1.8.0) platform (Caporaso et al., 2010) and R (v 3.4). An OTU table was built to store the abundance for each taxonomic units. The operational taxonomic unit threshold (c value) was set to 0.01% in QIIME with other parameters following a previous suggestion (Bokulich et al., 2013). All samples were rarefied to even depth of 11,000 reads for the diversity analyses. Beta diversity analysis was conducted by using weighted UniFrac distance metrics (Lozupone and Knight, 2005) and visualized by principal coordinate analysis (PCoA) (Ramette, 2007). The PERMANOVA analysis (permutational multivariate analysis of variance) was conducted between microbiome composition and environmental characteristics, such as sampling strategy, building age, building location, sex of occupants, wall surface type and cleaning frequency. This analysis was performed by adonis function of the vegan package in R. The microbiome similarity search was conducted on the Microbiome Search Engine (MSE, version 2.0, http://mse.single-cell.cn/) (Su et al., 2018), and the query samples were first pre-processed by Parallel-META 3 (Su et al., 2012; Su et al., 2013).

### Statistical analysis for health-associated microorganisms

The regression models were performed in STATA v15.0 (StataCorp LLC). The associations between relative abundance of airborne bacterial genera and rhinitis in last 12 months were calculated in a hierarchical logistic regression model, with the dormitory building as the second hierarchy. The model was adjusted for gender and smoking. The genera with a relative abundance less than 0.1% or with presence in less than 20 dormitory rooms were excluded. Therefore, the regression was conducted for 67 bacterial genera. The association between environmental characteristics and rhinitis-related bacterial species were calculated in a linear regression model.

## Results

### Demographic statistics

In this study, we used a multiplexed Illumina high-throughput sequencing technic to characterize culture-independent microbial composition in 86 randomly selected university dormitory rooms in 10 buildings from Shanxi University, China. Building 1-5 were from a new campus (LD), and building 6-10 were from an old campus (WY). The two campuses were 1.5 kilo meters away. A total of 357 students, of which 248 (69.4%) were females, participated in the health survey. The dormitory rooms, ranging in size from 12 to 23 m^2^, all featured natural ventilation through doors and windows.

Among the 357 students, 61 (17.1%) reported weekly rhinitis symptoms in the past 12 months were defined as having rhinitis. The prevalence of rhinitis symptoms showed no significant difference when comparing male and female students, smoking and non-smoking students and students living in new and old campuses (Chi-Square test, p = 0.25, 0.55 and 0.65, respectively).

### Sequencing statistics

The gel electrophoresis of negative control showed that our samples were not contaminated by laboratory microbes (**Supplementary Figure S1**). Chimeric and low-quality reads were filtered out before reads assembly and taxonomical classification. The number of quality filtered sequencing reads per sample ranged from 27,377 to 53,783 (interquartile range 32,710 – 41,377). The accumulation curve analysis suggested that first 20 settled air dust samples caught the majority of OTUs, suggesting that the sequencing depth is adequate to uncover a large proportion of low-frequency bacteria (**Supplementary Figure S2**). All samples were rarefied to an even depth of 27,000 reads, based on the sequencing depth of the shallowest sample, to ensure consistent representation across all samples in subsequent microbiome analyses.

### Bacterial diversity and composition

**Figure 1:**
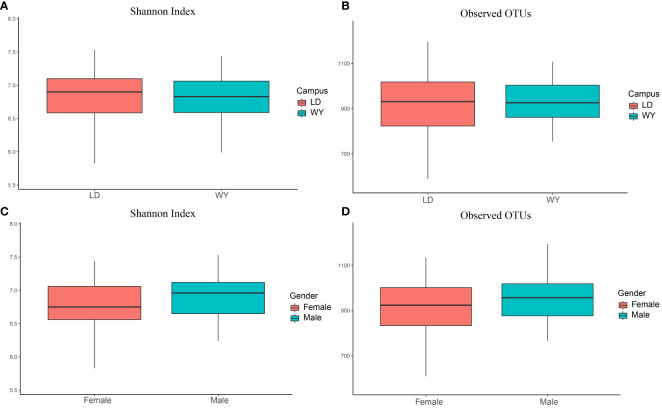
Bacterial alpha diversity between two campuses **(A, B)** and dormitory rooms for male and female students **(C, D)**. This box plot illustrates the distribution of alpha diversity. The line within each box represents the median value. The box represents the interquartile range (IQR), with the lower edge indicating the first quartile (Q1) and the upper edge representing the third quartile (Q3). The whiskers represent the smallest and largest values within 1.5 times the IQR from the lower and upper quartiles, respectively.

The number of observed OTUs and Shannon index for indoor bacteria were 904.4 ± 180.1 and 6.69 ± 0.95, respectively (mean ± standard deviation). We further compare the bacterial alpha diversity between two campuses and dormitory rooms for male and female students (**Figure 1**). Bacterial alpha-diversity did not differ between the two campuses (Student t test, observed OTUs, LD = 892.4 ± 220.8, WY = 918.2 ± 118.7, p = 0.51; Shannon index, LD = 6.59 ± 1.25, WY = 6.81 ± 0.38, p = 0.30) and dormitory rooms for male and female students (observed OTUs, male = 901.9 ± 226.2, female = 905.9 ± 146.7, p = 0.92; Shannon index, male = 6.67 ± 1.26, female = 6.70 ± 0.72, p = 0.88).

**Figure 2:**
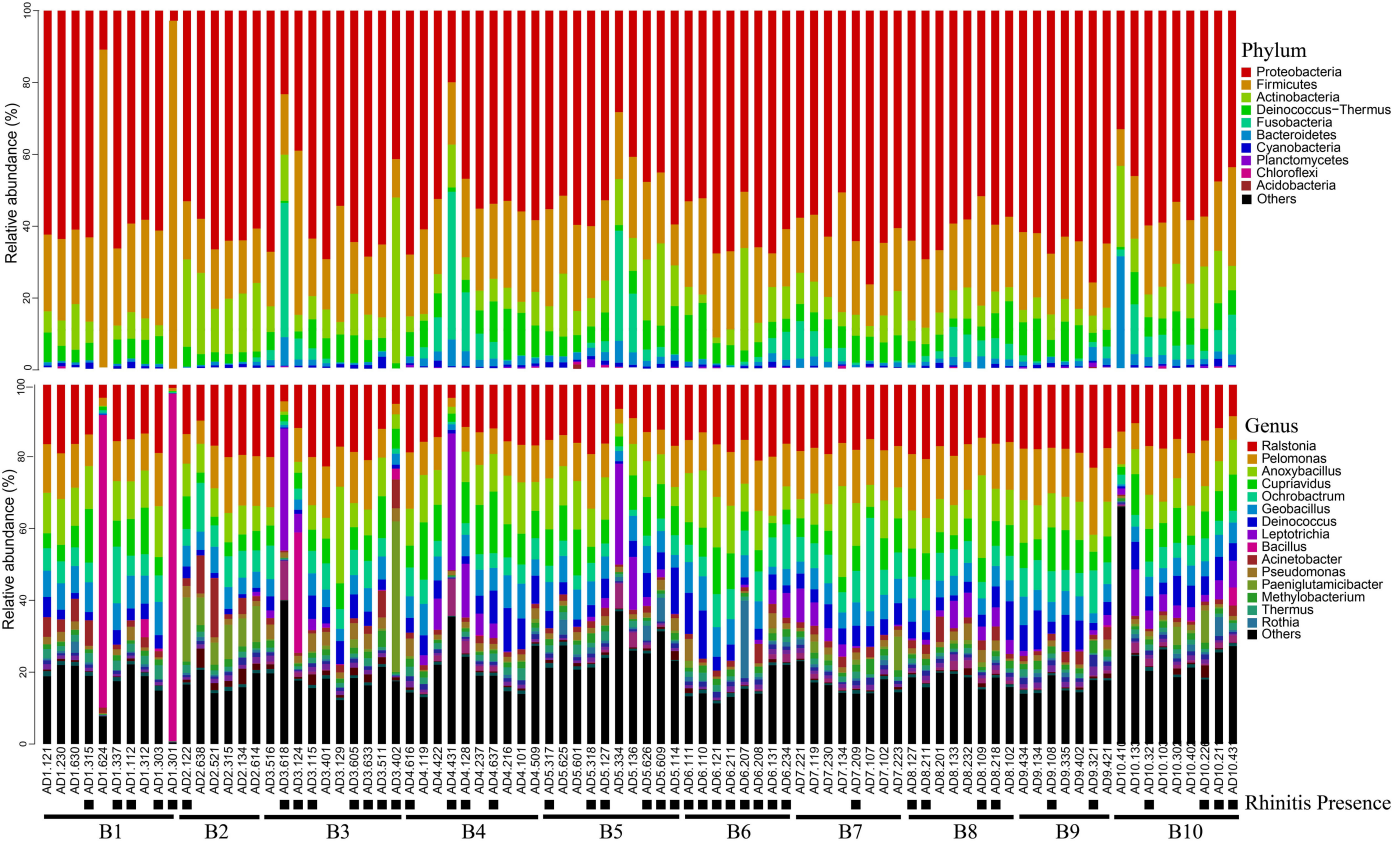
Bacterial relative abundance at phylum and genus level for settled air dust. The presence of rhinitis was shown below the room number. ‘B1’ is an abbreviation for Building 1.

**Figure 3:**
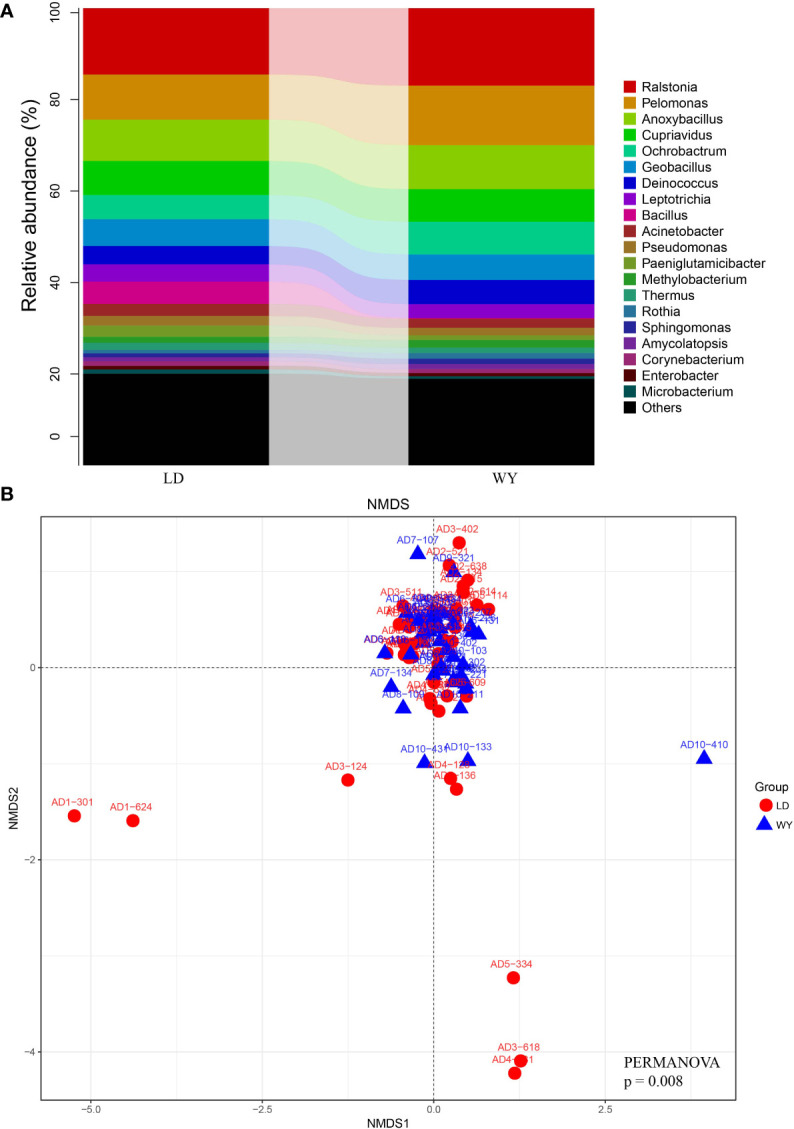
Microbial compositional variation between two campuses. **(A)** The relative abundance of bacterial taxa at the genus level for LD (new) and WY (old) campuses. **(B)** NMDS analyses of microbial composition based on weighted UniFrac distance. The ordination plot between NMDS1 and NMDS2 are displayed.

Relative abundance analysis showed that Proteobacteria accounted for 56.2% of all bacteria, followed by Firmicutes (21.5%), Actinobacteria (9.0%), Deinococcus-Thermus (6.0%), Fusobacteriia (3.9%) and Bacteroidetes (2.0%, **Figure 1A**). In total, 530 genera were identified with the mean of 184 genera detected (184.0 ± 32.5, mean ± standard deviation). The top genera were *Ralstonia* (15.6%) and *Pelomonas* (11.3%), followed by *Anoxybacillus* (9.3%), *Cupriavidus* (7.3%), *Ochrobactrum* (6.2%), *Geobacillus* (5.7%), *Deinococcus* (4.6%), *Leptotrichia* (3.4%), *Bacillus* (2.7%) and *Acinetobacter* (2.3%; **Figures 2**, **3A**). The overall indoor microbial composition was found to significantly differ between the two campuses (PERMANOVA, p = 0.008). However, according to the NMDS analysis, the observed variation between the new and old campuses appeared to be primarily attributed to a few dormitories in the new campus (**Figure 3B**). For instance, dormitory rooms 301 and 624 in Building 1 exhibited a microbial community dominated by *Bacillus* (**Figures 2**, **3B**). In contrast, most other dormitories in the new and old campus displayed similar microbial compositions, predominantly characterized by *Ralstonia*, *Pelomonas*, and *Anoxybacillus* (**Figure 3A**).

We also explored that whether the dormitory microbiome composition was similar to other previous reported microbiome composition from various environmental niches, by searching against the Microbiome Search Engine (MSE) (Su et al., 2018). More than 60% of settled air dust samples from dormitory rooms were best matched with the building environment, and approximately 20% were best matched with the human nasopharynx samples. The results fit with our expectation, indicating that the dormitory microbiome in Shanxi University is a representative microbiome composition for the indoor environment.

### Health association between indoor bacteria and rhinitis

Association between microbial richness (number of observed OTUs) and rhinitis were assessed (**Table 1**). The overall bacterial richness was not associated with rhinitis (p = 0.37). The analysis was further conducted at the class level. Taxa richness in Actinobacteria and Fusobacteria was negatively associated with rhinitis (p = 0.02 and 0.03), indicating that exposing a higher diverse set of Actinobacteria and Fusobacteriia may reduce the occurrence of rhinitis. Taxa richness in Bacteroidia showed marginally negative association with rhinitis (p = 0.07). Taxa richness in Alphaproteobacteria and Betaproteobacteria showed a slight trend of positive association with rhinitis (p = 0.12 and 0.18).

**Table 1:** Association between bacterial richness (represented as the number of observed OTUs) and rhinitis symptoms among students (N = 347).

Kingdom	Phylum	Class	Number of OTUs, Median (Q1,Q3)	Rhinitis	
				OR (95% CI)	p-value
Bacteria	Proteobacteria	Alphaproteobacteria	134 (117,160)	1.28 (0.94,1.76)	0.12
		Betaaproteobacteria	249 (208,297)	1.14 (0.94,1.39)	0.18
		Gammaproteobacteria	269 (209,329)	0.82 (0.42,1.59)	0.56
	Bacteroidetes	Bacteroidia	33 (27,41)	0.88 (0.77,1.01)	0.07
	Actinobacteria	Actinobacteria	120 (103,143)	**0.69 (0.51,0.95)**	**0.02**
	Fusobacteria	Fusobacteriia	98 (83-125)	**0.88 (0.79,0.99)**	**0.03**
	Firmicutes	Bacilli Clostridia	165 (120,196) 42 (30-50)	1.14 (0.87,1.50) 1.17 (0.94,1.46)	0.34 0.16
	Deinococcus-Thermus	Deinococci	50 (35,79)	1.03 (0.90,1.18)	0.65
Total bacterial OTUs			1,205 (1,061,1,263)	1.00 (1.00,1.01)	0.37

Taxonomical classes with a total number of OTUs > 50 among all samples was included in the analysis. Cyanobacteria were not resolved at the class level, and thus the calculation was conducted at the phylum level. Odds ratio (OR) and 95% confidence interval (CI) were calculated by 2-level logistic regression models adjusted for gender and smoking. The odds ratio was calculated for 100 OTU increase. P values < 0.05 were formatted with bold font.

To find the rhinitis-associated microorganisms, we conducted a logistic regression analysis between the abundance of indoor microorganisms and the self-administered health data of rhinitis in the dormitory occupants. For rhinitis, all genera with an average relative abundance higher than 0.1% and presence in more than 20 rooms were included for the association analysis. Thirteen airborne bacterial genera were observed positively or protectively associated with rhinitis (p<0.01) in last 12 months (**Table 2**). The results are similar to the richness association analysis: microbial taxa that were negatively associated with occurrence of rhinitis were mainly from Actinobacteria (*Actinomyces*), Bacteroidetes (*Prevotella, Capnocytophaga*), and Fusobacteriia (*Fusobacterium*), whereas microbial genera that were positively associated with rhinitis were mainly from the class of Alphaproteobacteria and Betaproteobacteria (*Sphingomonas, Caulobacter*, uncharacterized *Caulobacteraceae* and *Comamonadaceae*). Overall, the results indicate that microbial taxa from different taxonomy could have different health effects for rhinitis.

**Table 2:** Association between indoor bacterial abundance and rhinitis symptoms among students (N = 347).

Phylum	Class	Genus	Relative abundance (%)	OR (95% CI)	p-value	False discovery rate
Actinobacteria	Actinobacteria	*Actinomyces*	0.11	0.09 (0.01, 0.5)	0.006	0.047
Bacteroidetes	Sphingobacteriia	*Sediminibacterium*	0.50	2.5 (1.39, 4.48)	0.002	0.045
	Bacteroidia	*Prevotella*	0.19	0.35 (0.17, 0.74)	0.006	0.047
	Flavobacteriia	*Capnocytophaga*		0.2 (0.06, 0.66)	0.008	0.047
Deinococcus-Thermus	Deinococci	*Thermus*	1.40	1.60 (0.06, 39.86)	0.002	0.045
Firmicutes	Negativicutes	*Selenomonas*	0.30	0.48 (0.28, 0.82)	0.008	0.047
Fusobacteria	Fusobacteriia	*Fusobacterium*	0.33	0.52 (0.32, 0.83)	0.006	0.047
Planctomycetes	Phycisphaerae	Phycisphaeraceae SM1A02	0.16	8.79 (1.79, 43.1)	0.007	0.047
Proteobacteria	Alphaproteobacteria	*Sphingomonas*	0.98	2.14 (1.26, 3.64)	0.005	0.047
		*Caulobacter*	0.63	3.45 (1.39, 8.53)	0.007	0.047
		uc Caulobacteraceae	0.13	3.22 (0.24, 43.36)	0.009	0.047
	Betaproteobacteria	uc Comamonadaceae	1.11	2.02 (0.004, 967.0)	0.002	0.045
	Gammaproteobacteria	*Aggregatibacter*	0.14	0.22 (0.07, 0.69)	0.009	0.047

The odds ratio and 95% confidence interval (CI) were calculated by 2-level logistic regression models adjusted for gender and smoking. The regression was conducted for genus presented in at least 20 dormitory rooms with mean relative abundance > 0.1%. In total, 67 bacterial genera were analyzed. Taxonomic information of the associated microbes is presented. Only significantly associated species (p < 0.01) are presented in the table. The false discovery rate (FDR) was calculated by Benjamini-Hochberg (BH) procedure. Positive associations were coded with red color, and negative associations were coded with green color.

### Association between environmental characteristics and rhinitis-related taxa

The association between environmental characteristics and potential protective microbial taxa of rhinitis were assessed by linear regression model (**Table 3**). Living on a higher floor level was associated with an increased abundance of three protective taxa, including *Prevotella*, *Aggregatibacter*, and *Actinomyces* (p<0.01). Having curtain indoor was negatively associated with two protective taxa, including *Fusobacterium* and *Prevotella* (p<0.01). PM_2.5_ concentration was positively associated with five protective taxa, including *Selenomonas*, *Prevotella*, *Aggregatibacter*, *Capnocytophaga*, and *Actinomyces* (p<0.01).

**Table 3:** Associations between environmental characteristics and the abundance of potential protective microbial taxa of rhinitis in university dormitory rooms. Bivariate linear regression was used to calculate the beta coefficient and 95% confidence interval (CI).

Environmental characteristics	Fusobacteriia	Negativicutes	Bacteroidia	Gammaproteobacteria	Flavobacteriia	Actinobacteria
	*Fusobacterium*	*Selenomonas*	*Prevotella*	*Aggregatibacter*	*Capnocytophaga*	*Actinomyces*
Old building age	-0.15(-0.28,-0.03) 0.018	-0.20(-0.13,0.09) 0.716	-0.08(0.16,-0.0005) 0.049	-0.05(-0.11,0.002) 0.057	0.001(-0.05,0.05) 0.954	-0.02(-0.05,0.01) 0.209
High floor level	0.10(0.03,0.16) 0.005	0.70(0.01,0.13) 0.015	0.06(0.02,0.10) **0.007**	0.04(0.01,0.07) **0.006**	0.03(0.00002,0.05) 0.050	0.02(0.006,0.4) **0.007**
Orientation	0.08(0.01,0.14) 0.023	0.06(0.004,0.11) 0.037	-0.02(-0.02,0.065) 0.244	0.03(0.006,0.06) 0.018	0.03(0.004,0.05) 0.023	0.02(0.008,0.04) **0.002**
Wall surface type	0.30(-0.001,0.53) 0.054	0.24(0.03,0.46) 0.028	0.20(0.01,0.32) 0.036	0.12(0.02,0.23) 0.021	0.10(0.009,0.19) 0.033	0.06(0.01,0.12) 0.019
Having curtain indoor	-0.20(-0.28,-0.03) **0.018**	-0.02(-0.18,0.09) 0.716	-0.08(-0.16,-0.0005) **0.049**	-0.05(-0.11,0.002) 0.057	0.001(-0.05,0.05) 0.954	-0.019(-0.05,0.01) 0.209
High frequency of curtain cleaning	-0.32(-0.78,0.15) 0.177	-0.30(-0.65,0.11) 0.155	-0.20(-0.44,0.10) 0.220	-0.12(-0.30,0.06) 0.192	-0.106(-0.27,-0.06) 0.195	-0.08(-0.17,0.009) 0.078
Having plant indoor	-0.05(-0.35,0.24) 0.725	-0.02(-0.26,0.22) 0.887	-0.007(-0.18,0.17) 0.934	-0.0009(-0.12,0.11) 0.988	0.001(-0.10,0.10) 0.979	0.005(-0.05,0.06) 0.874
Indoor CO_2_ concentration	-0.1(-0.20,0.005) **0.0001**	-0.04(-0.1,0.05) 0.391	-0.05(-0.1,0.01) 0.111	-0.03(-0.08,0.01) 0.165	-0.02(-0.06,0.02) 0.392	-0.01(-0.04,0.02) 0.429
Indoor PM_2.5_ concentration	-0.46(0.27,0.66) **0.0001**	0.31(0.15,0.48) **0.0001**	0.26(0.13,0.38) **0.0001**	0.18(0.10,0.27) **0.0001**	0.13(0.05,0.20) **0.001**	0.10(0.06,0.15) **0.0001**

Associations with p < 0.01 are highlighted in bold. P-values are indicated below the corresponding beta coefficient and 95% CI.

Similar analysis was conducted between environmental characteristics and potential risk microbial taxa of rhinitis (**Table 4**). Living in old buildings was associated with an increased abundance of four risk Proteobacteria, including uc Comamonadaceae, uc Caulobacteraceae, *Sphingomonas*, and *Caulobacter* (p<0.01). Living in higher floor level was associated with a decreased abundance of two risk taxa, including *Sphingomonas* and *Sediminibacter* (p<0.01). Having curtain indoor was positively associated with four risk Protecbacteria, including uc Comamonadaceae, uc Caulobacteraceae, *Sphingomonas*, and *Caulobacter* (p<0.01). Indoor CO_2_ concentration, indicative of higher building confinement and reduced ventilation, was positively associated with three risk Protecbacteria, including uc Comamonadaceae, uc Caulobacteraceae, and *Sphingomonas* (p<0.01). PM_2.5_ concentration was negatively associated with six risk taxa, including uc Comamonadaceae, uc Caulobacteraceae, *Sphingomonas*, Caulobacter, Phycisphaeraceae SM1A02, and *Sediminibacter* (p<0.01).

**Table 4:** Associations between environmental characteristics and the abundance of potential risk microbial taxa of rhinitis in university dormitory rooms.

Environmental characteristics	Betaproteobacteria	Alphaproteobacteria			Deinococci	Phycisphaerae	Sphingobacteriia
	uc Comamonadaceae	uc Caulobacteraceae	*Sphingomonas*	*Caulobacter*	*Thermus*	Phycisphaeraceae SM1A02	*Sediminibacter*
Old building age	0.05(0.03,0.07) **0.001**	0.03(0.02,0.04) **0.001**	0.38(0.28,0.48) **0.001**	0.12(0.07,0.17) **0.001**	-0.04(-0.05,-0.02) **0.001**	0.05(0.01,0.09) 0.014	-0.03(-0.15,0.09) 0.604
High floor level	-0.01(-0.02,-0.003) 0.012	-0.004(-0.01,0.001) 0.138	-0.13(-0.19,-0.08) **0.001**	-0.02(-0.05,0.004) 0.090	-0.008(-0.02,-0.0001) 0.047	-0.007(-0.03,0.01) 0.530	-0.16(-0.22,-0.09) **0.001**
Wall surface type	-0.05(-0.09,-0.002) 0.039	-0.02(-0.04,0.002) 0.079	-0.14(-0.30,0.01) 0.071	-0.07(-0.15,0.02) 0.130	-0.02(-0.06,0.01) 0.228	0.004(-0.03,0.04) 0.788	0.17(-0.009,0.35) 0.062
Having curtain indoor	0.05(0.03,0.07) **0.001**	0.03(0.02,0.04) **0.001**	0.38(0.29,0.48) **0.001**	0.12(0.07,0.170) **0.001**	-0.04(-0.05,-0.02) **0.001**	0.05(0.01,0.09) 0.014	-0.32(-0.15,0.09) 0.604
High frequency of curtain cleaning	0.09(0.02,0.17) 0.019	0.02(-0.01,0.06) 0.187	0.11(-0.16,0.39) 0.404	0.05(-0.10,0.20) 0.525	-0.02(-0.09,0.04) 0.488	0.02(-0.04,0.07) 0.554	-0.35(-0.66,-0.04) 0.029
Having plant indoor	0.03(-0.02,0.08) 0.240	0.03(0.007,0.05) 0.011	0.19(0.03,0.36) 0.024	0.003(-0.09,0.10) 0.945	0.02(-0.02,0.06) 0.447	0.03(-0.01,0.06) 0.104	0.13(-0.07,0.33) 0.190
Indoor CO_2_ concentration	0.02(0.007,0.04) **0.003**	0.02(0.007,0.03) **0.0001**	0.1(0.06,0.2) **0.001**	-0.02(-0.03,-0.008) **0.001**	0.02(-0.03,0.06) 0.65	-0.1(-0.20,0.005) 0.062	-0.2(-0.3,-0.07) **0.0001**
Indoor PM_2.5_ concentration	-0.07(-0.09,-0.04) **0.0001**	-0.02(-0.04,-0.006) **0.009**	-0.32(-0.49,-0.16) **0.0001**	-0.03(-0.06,-0.01) **0.005**	-0.19(-0.27,-0.11) **0.0001**	0.18(0.10,0.27) **0.0001**	-0.35(-0.55,-0.16) **0.0001**

Bivariate linear regression was used to calculate the beta coefficient and 95% confidence interval (CI). Associations with p < 0.01 are highlighted in bold. P-values are indicated below the corresponding beta coefficient and 95% CI.

We also conducted association analysis between these environmental characteristics and the occurrence of rhinitis. However, none of these environmental characteristics were significantly associated with rhinitis (p>0.05).

## Discussion

### Strengths and limitations of the study

This study boasts several strengths that enhance its contribution to the field. Firstly, it is the first study to investigate the indoor microbiome and its relationship with rhinitis among young adults living in a university dormitory. This novel setting provides unique insights into the microbiome’s role in health and disease among this population. Secondly, the study includes a wide collection of environmental characteristics. This wide-ranging data allows for an in-depth analysis of the associations between these characteristics and rhinitis-related taxa, providing potential intervention strategies for future research. Thirdly, this study utilized settled air dust collected in Petri dishes, rather than the commonly used vacuum dust. Traditional vacuum dust sampling primarily targets floor and table surfaces, which may not effectively represent airborne microorganisms that people are exposed to through inhalation. This point is supported by a study conducted by Hyytiäinen et al. (2018), which reported significant differences in the microbial composition between floor vacuum dust and air dust at varying heights. To address this limitation, we employed open Petri dishes hung at a height of 1.2-1.5 meters in the dormitory rooms. This method enables the capture of settled air dust over seven days, providing a more representative sample of the airborne microorganisms likely to be inhaled by individuals.

However, the study has certain limitations. Firstly, we used amplicon sequencing, which, while effective for identifying taxonomic diversity, does not allow for functional potential and species-level characterization (Fu et al., 2022). Secondly, the cross-sectional nature of our study inherently limits our ability to determine causality. While our current cross-sectional study highlights correlations between indoor microbiome compositions and rhinitis symptoms, it inherently limits our ability to establish causality and the temporal order of these associations. Recognizing this limitation, we emphasize the importance of longitudinal studies for future research. Such studies would enable tracking of microbiome changes over time in relation to the onset and progression of rhinitis, thereby potentially uncovering causal relationships. Our findings, though significant, are preliminary and underscore the need for long-term investigations to understand better the dynamics of microbiome-rhinitis interactions and to establish causative mechanisms. Third, the Petri dish method we employed for sampling settled air dust has inherent limitations. The approach may be subject to variability introduced by factors such as air movement, door openings/closings, and occupants moving around in the dormitories. However, this method also has its strength which can effectively captures a comprehensive sample of settled air dust indicative of cumulative exposures over a week, providing a realistic representation of the indoor environment’s microbiome to which students are routinely exposed. Fourth, we acknowledge that the number of participants in our study, though valuable for preliminary insights, is relatively limited. The confined sample size might constrain the breadth and generalizability of our findings. Fifth, by focusing exclusively on dormitories, our study provides a distinct snapshot, which may not capture the intricacies of broader age groups or diverse living conditions. While our results present important trends within the context of this specific setting, they might not be directly applicable to other environments or populations. Future large-scale studies or meta-analyses that incorporate a diverse range of indoor settings and larger participant groups will be crucial to discern a general pattern between indoor microbiomes and their health impacts. Sixth, the dust samples were exclusively collected during November and December, which are characterized by colder and potentially drier conditions. This narrow seasonal timeframe does not allow for the evaluation of seasonal variations in the indoor microbiome and its potential influence on rhinitis symptoms. Therefore, our results provide insights specific to this colder period and might not be representative of the entire year. Future studies spanning multiple seasons will be essential to capture a comprehensive understanding of the year-round relationship between the indoor microbiome and rhinitis symptoms.

### Potential protective and risk microbial taxa of rhinitis

In our study, we identified several microbial taxa associated with rhinitis, highlighting the importance of understanding the role of the microbiome in the pathogenesis of this condition. Of these taxa, only one has been directly linked to rhinitis in previous research. A previous epidemiological survey conducted in preschools in Taiyuan, China, reported that *Prevotella* was protectively (negatively) associated with rhinitis (Sun et al., 2022a). In addition, *Prevotella* has been reported to have protective effects against gastrointestinal diseases (Tett et al., 2021), indicating that these bacteria might play a beneficial role in human health, potentially by modulating immune responses. Other genera identified in our study have connections to various human health conditions. For instance, *Sphingomonas paucimobilis* has been implicated in nosocomial infections and inflammation (Ryan and Adley, 2010). While such conditions are distinct from rhinitis, the potential inflammatory characteristics of these bacteria might contribute to the inflammatory symptoms observed in rhinitis.

A majority of the microbial taxa identified in this study has not been reported in previous indoor microbiome and rhinitis/asthma studies (Fu et al., 2020b; Fu et al., 2022; Fu et al., 2023a; Sun et al., 2023; Zhang et al., 2023b). Several reasons could underlie this discrepancy. First, the diversity of environmental microbes is extraordinary. Earth’s microbial species count is estimated to exceed 1 trillion (Locey and Lennon, 2016). Within individual rooms, more than 10,000 distinct microbial species can be identified (Brooks et al., 2014; Barberán et al., 2015a), a count that far surpasses the diversity found in the human gut, which typically harbors about 160 species (Qin et al., 2010). Second, indoor microbiomes has pronounced geographical distinctions (Amend et al., 2010; Barberán et al., 2015a; Barberán et al., 2015b; Fu et al., 2020a). Different geographical locations often have unique microbial compositions, with each hosting a distinctive set of health-related microbes. A primary reason for such strong variation in indoor microbiomes is the immense geographical diversity of soil and air microbiota (O'Brien et al., 2016; Fierer, 2017; Leung et al., 2021). A plethora of these environmental microorganisms, originating from outdoor spaces, can infiltrate indoor environments via air and other transmission routes, diversely impact the indoor microbial landscape and occupant health. Third, various environmental parameters, such as relative humidity, outdoor pollutants, local vegetation, and specific indoor characteristics, play pivotal roles in shaping the indoor microbiome (Fu et al., 2020a; Fu et al., 2022). The intricate relationship between these environmental factors and the indoor microbiome introduces added complexity when trying to identify health-related microorganisms, particularly when the environmental factors themselves are also associated with rhinitis or asthma. In such scenarios, employing causal mediation analysis could be an effective strategy to unravel the complex interconnections among various factors.

### Environmental characteristics associated with protective and risk taxa of rhinitis

Environmental characteristics are a crucial aspect of indoor microbiota composition and consequently can affect the prevalence and exacerbation of health conditions such as rhinitis. Our results demonstrated specific relationships between these characteristics and both protective and risk microbial taxa associated with rhinitis.

Living at higher floor levels was associated with an increased abundance of protective taxa including *Prevotella* and *Aggregatibacter*. This is possibly due to less ground-level dust and outdoor risk microbial intrusion, thus fostering an environment that enhances the relative abundance of these protective taxa (Adams et al., 2013). Additionally, we found that indoor PM_2.5_ concentration was positively associated with five protective taxa, including *Selenomonas*, *Prevotella*, *Aggregatibacter*, *Capnocytophaga*, and *Actinomyces*. This result aligns with previous research indicating that fine particulate matter can serve as a vehicle for indoor airborne microorganisms, thus influencing microbial diversity and composition (Fu et al., 2021b; Sun et al., 2022a). It’s also possible that in environments with low PM_2.5_ levels, the scarcity of nutrients may lead to heightened microbial competition. Under these conditions, only the most competitive or adaptive microorganisms might dominate, potentially explaining the observed microbial associations in our study.

Conversely, having curtains indoors was negatively associated with two protective taxa, including *Fusobacterium* and *Prevotella*. Curtains, being fabric materials, can trap and accumulate dust and may alter the indoor microbial community by providing habitats for different microorganisms, potentially affecting the balance between protective and risk taxa. A recent study in commercial aircraft showed that compared to cabins with leather surfaces, cabins with textile surfaces were enriched with microbial virulence factors, facultative pathogens, and risk chemicals such as pesticides and detergents (Fu et al., 2023b).

Regarding risk taxa, we found that living in older buildings and having curtains indoors increased the abundance of risk Proteobacteria, possibly due to higher rates of potential risk microbial accumulation in these environments. Additionally, higher indoor CO_2_ concentration, indicative of poor ventilation, was positively associated with risk Proteobacteria, suggesting that building design and maintenance practices impacting ventilation are crucial to managing the microbiota balance (Kembel et al., 2012; Meadow et al., 2014).

Overall, these observations suggest that strategies to manage indoor environments, such as enhancing ventilation, reducing fabric materials, and maintaining cleaner living spaces, especially in older buildings, could be effective in controlling the abundance of risk taxa. These findings collectively indicate that modification of indoor environments could potentially be a strategic approach in controlling and managing rhinitis by manipulating the balance of protective and risk microbial taxa. Future research should focus on testing the effectiveness of such environmental modifications in reducing the prevalence and severity of rhinitis, as well as other respiratory diseases, in real-world settings.

## Data availability statement

The data presented in the study are deposited in the QIITA repository, accession number “12841”.

## Ethics statement

The studies involving humans were approved by Medical Ethics Committee of Fudan University, Shanghai, China. The studies were conducted in accordance with the local legislation and institutional requirements. The participants provided their written informed consent to participate in this study.

## Author contributions

XF: Data curation, Project administration, Writing – original draft. AS: Data curation, Writing – review & editing. DN: Writing – review & editing, Project administration. QC: Writing – review & editing, Methodology. YX: Writing – review & editing. XZ: Writing – review & editing, Investigation. YS: Data curation, Project administration, Writing – original draft.
