# Supplementary material for: Exploring the role of indoor microbiome and environmental characteristics in rhinitis symptoms among university students
Source: Front Microbiomes. 2024 Feb 16;3:1277177. doi: 10.3389/frmbi.2024.1277177 (PMC12993640; doi:10.3389/frmbi.2024.1277177)
Supplement: Supplementary file 1 [file DataSheet_1.docx]

Supplementary Figures


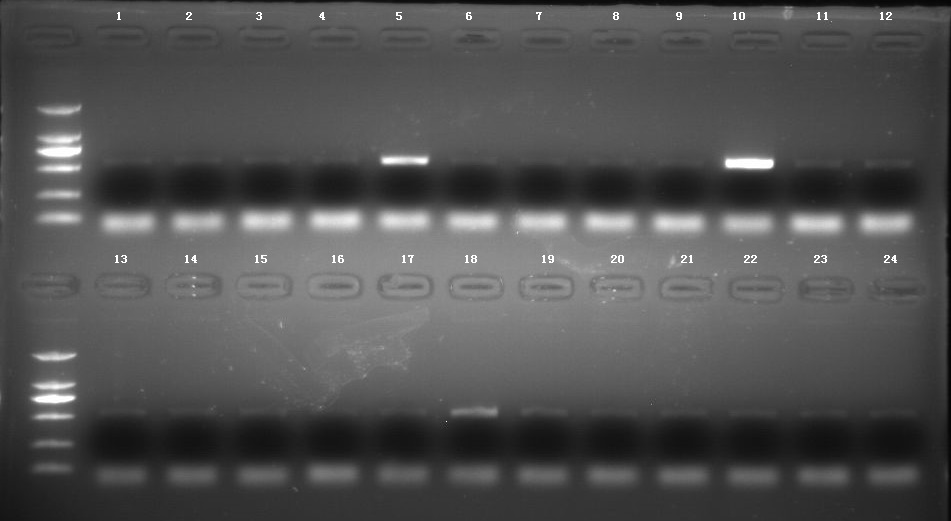

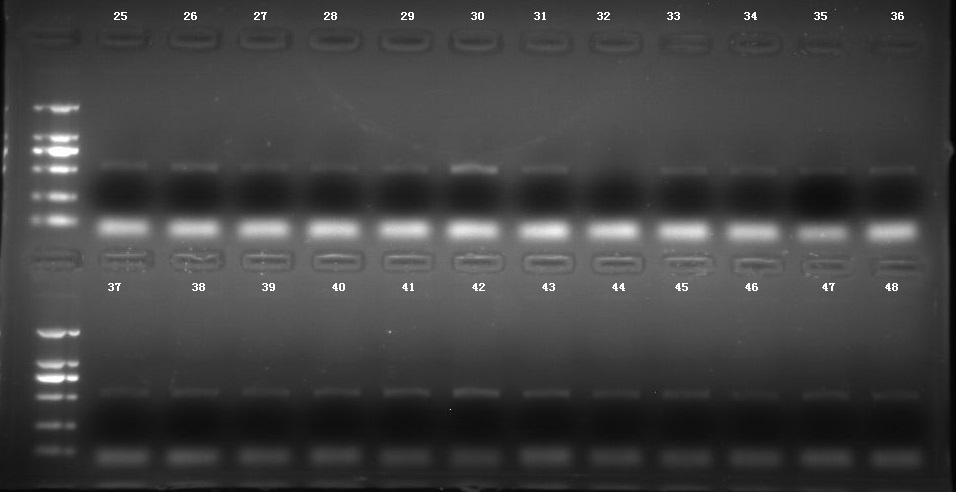

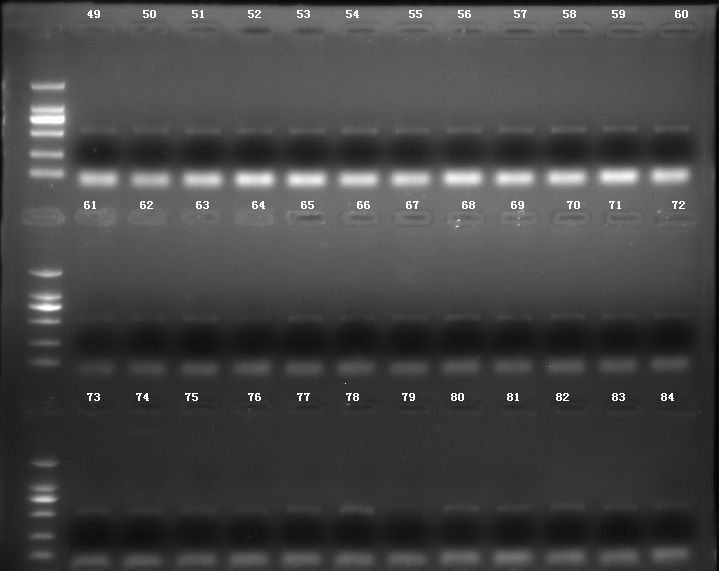

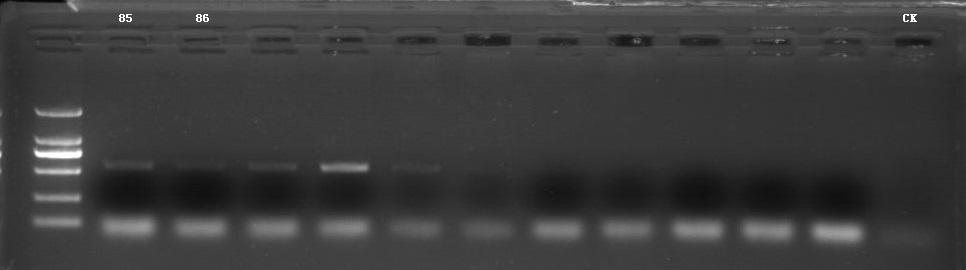


Figure S1. Gel electrophoresis of amplified 16S rRNA in the air dust samples and a negative control marked as CK.


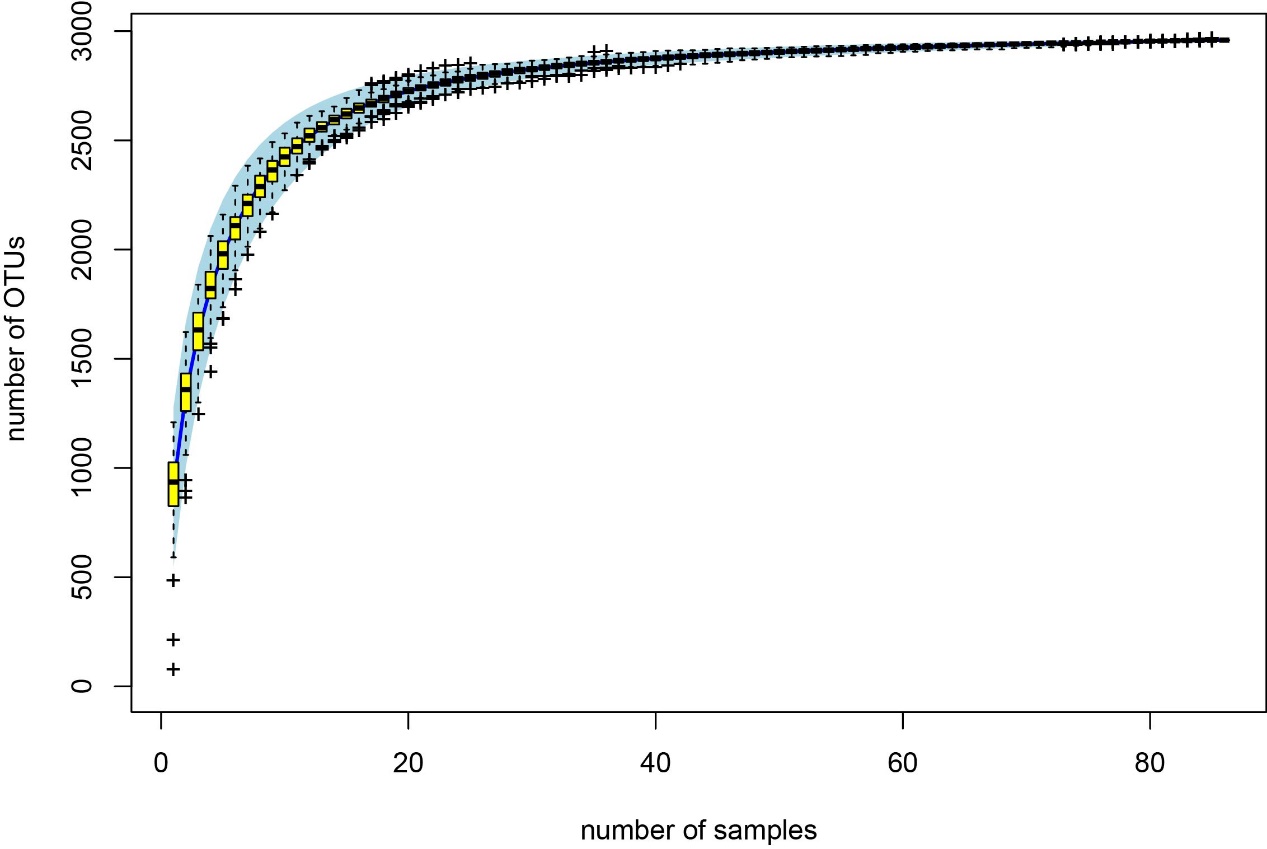


Figure S2. Species accumulation curve for settled air dust. The dark black line is the median species accumulation curve from 100 random permutations of the data. The yellow box represents Q1 (25%) and Q3 (75%) quartile. The top whisker represents Q3 + 1.5*IQR (interquartile range), and the bottom whisker represents Q1 – 1.5*IQR, and the “+” symbol represents outliers in the analysis. The shaded light blue area represents 2 times the standard deviations.
