# Supplementary material for: Exploring the role of indoor microbiome and environmental characteristics in rhinitis symptoms among university students
Source: Front Microbiomes. 2024 Feb 16;3:1277177. doi: 10.3389/frmbi.2024.1277177 (PMC12993640; doi:10.3389/frmbi.2024.1277177)
Supplement: Supplementary file 2 [file Table_1.docx]

Table S1. Microbiome compositional similarity analysis. MNS stands for Microbiome Novelty Score.

| **SampleID** | **MNS** | **First_match_sample** | **Second_match_sample** | **Third_match_sample** | **Overall habitat prediction** |
| --- | --- | --- | --- | --- | --- |
| AD10103 | 0.157 | other_humanbodysite | non_mammal_animal | soil | nasopharyngeal |
| AD10133 | 0.149 | other_humanbodysite | building | skin | building |
| AD10211 | 0.132 | other_humanbodysite | building | food | building |
| AD10226 | 0.129 | skin | building | non_mammal_animal | skin |
| AD10302 | 0.139 | skin | non_mammal_animal | building | bird |
| AD10321 | 0.142 | other_humanbodysite | skin | sand | nasopharynx |
| AD10402 | 0.146 | building | building | building | building |
| AD10410 | 0.154 | gut | gut | gut | gut |
| AD10431 | 0.17 | building | other_humanbodysite | building | building |
| AD1112 | 0.153 | skin | building | other_humanbodysite | building |
| AD1121 | 0.145 | building | building | building | building |
| AD1230 | 0.141 | building | building | building | building |
| AD1301 | 0.065 | mammal_animal | mammal_animal | soil | mouse |
| AD1303 | 0.155 | building | sand | skin | soil |
| AD1312 | 0.156 | building | non_mammal_animal | skin | building |
| AD1315 | 0.146 | other_humanbodysite | skin | building | nasopharynx |
| AD1337 | 0.161 | building | building | sand | building |
| AD1624 | 0.094 | other | other | marine | anthropogenic_environment |
| AD1630 | 0.133 | skin | building | building | building |
| AD2122 | 0.131 | building | building | building | building |
| AD2134 | 0.126 | skin | skin | building | skin |
| AD2315 | 0.135 | other_humanbodysite | skin | non_mammal_animal | bird |
| AD2521 | 0.128 | skin | skin | skin | skin |
| AD2614 | 0.135 | skin | non_mammal_animal | food | bird |
| AD2638 | 0.119 | building | building | skin | building |
| AD3115 | 0.144 | skin | non_mammal_animal | other_humanbodysite | bird |
| AD3124 | 0.157 | building | building | building | building |
| AD3129 | 0.152 | other_humanbodysite | building | non_mammal_animal | nasopharynx |
| AD3401 | 0.131 | non_mammal_animal | other_humanbodysite | skin | vulture |
| AD3402 | 0.114 | building | building | food | building |
| AD3511 | 0.132 | building | building | skin | building |
| AD3516 | 0.149 | non_mammal_animal | skin | building | buffer |
| AD3605 | 0.137 | skin | non_mammal_animal | building | bird |
| AD3618 | 0.146 | skin | skin | skin | skin |
| AD3633 | 0.132 | non_mammal_animal | other_humanbodysite | skin | bird |
| AD4101 | 0.147 | other_humanbodysite | building | skin | soil |
| AD4119 | 0.14 | non_mammal_animal | other_humanbodysite | other | vulture |
| AD4128 | 0.185 | non_mammal_animal | skin | non_mammal_animal | bird |
| AD4216 | 0.148 | building | skin | building | building |
| AD4237 | 0.149 | skin | sand | building | filtration_sand |
| AD4422 | 0.158 | other_humanbodysite | skin | sand | soil |
| AD4431 | 0.147 | oral | skin | skin | skin |
| AD4509 | 0.142 | building | skin | non_mammal_animal | building |
| AD4616 | 0.142 | other_humanbodysite | building | sand | building |
| AD4637 | 0.149 | building | skin | skin | skin |
| AD5114 | 0.132 | non_mammal_animal | building | skin | bird |
| AD5127 | 0.137 | skin | other_humanbodysite | building | building |
| AD5136 | 0.156 | building | other_humanbodysite | building | building |
| AD5317 | 0.161 | other_humanbodysite | skin | non_mammal_animal | bird |
| AD5318 | 0.143 | skin | sand | other_humanbodysite | soil |
| AD5334 | 0.152 | skin | building | building | skin |
| AD5601 | 0.135 | soil | sand | soil | soil |
| AD5609 | 0.134 | building | skin | building | building |
| AD5625 | 0.125 | building | food | other_humanbodysite | building |
| AD5626 | 0.131 | building | food | skin | building |
| AD6110 | 0.156 | building | sand | skin | soil |
| AD6111 | 0.143 | skin | building | non_mammal_animal | building |
| AD6121 | 0.148 | other_humanbodysite | building | soil | nasopharynx |
| AD6131 | 0.142 | skin | building | building | building |
| AD6207 | 0.124 | building | food | building | building |
| AD6208 | 0.14 | other_humanbodysite | building | non_mammal_animal | nasopharynx |
| AD6211 | 0.144 | other_humanbodysite | building | non_mammal_animal | nasopharynx |
| AD6234 | 0.15 | building | skin | building | building |
| AD7102 | 0.139 | other_humanbodysite | other_humanbodysite | building | nasopharynx |
| AD7107 | 0.143 | mammal_animal | building | non_mammal_animal | building |
| AD7119 | 0.143 | other_humanbodysite | skin | building | building |
| AD7134 | 0.158 | building | sand | soil | soil |
| AD7209 | 0.147 | other_humanbodysite | building | skin | nasopharynx |
| AD7221 | 0.153 | skin | non_mammal_animal | other_humanbodysite | bird |
| AD7223 | 0.142 | other_humanbodysite | other_humanbodysite | building | nasopharynx |
| AD7230 | 0.146 | other_humanbodysite | building | skin | nasopharynx |
| AD8102 | 0.15 | other_humanbodysite | building | sand | nasopharynx |
| AD8109 | 0.144 | skin | building | building | building |
| AD8127 | 0.148 | other_humanbodysite | building | soil | nasopharynx |
| AD8133 | 0.15 | skin | sand | building | soil |
| AD8201 | 0.142 | other_humanbodysite | building | sand | building |
| AD8211 | 0.139 | other_humanbodysite | other_humanbodysite | soil | nasopharynx |
| AD8218 | 0.145 | other_humanbodysite | sand | soil | soil |
| AD8232 | 0.148 | skin | non_mammal_animal | building | building |
| AD9108 | 0.137 | other_humanbodysite | building | skin | nasopharynx |
| AD9134 | 0.151 | other_humanbodysite | building | non_mammal_animal | nasopharynx |
| AD9321 | 0.141 | other_humanbodysite | building | non_mammal_animal | soil |
| AD9335 | 0.142 | other_humanbodysite | building | skin | nasopharynx |
| AD9402 | 0.142 | other_humanbodysite | skin | building | nasopharynx |
| AD9421 | 0.136 | other_humanbodysite | skin | building | nasopharynx |
| AD9434 | 0.149 | other_humanbodysite | building | sand | soil |
